# Bacterial pathogens causing skin and soft tissue infections and antibiotic susceptibility in South Asia: a scoping review protocol

**DOI:** 10.1186/s13643-025-02885-1

**Published:** 2025-07-04

**Authors:** Rajalakshmi Rajendran, Shravya Chitrapady, Haritha K., Tejashree M U, Jayaraj Mymbilly Balakrishnan, Girish Thunga

**Affiliations:** 1 Department of Pharmacy Practice, Manipal College of Pharmaceutical Sciences, Manipal Academy of Higher Education, Manipal, Karnataka 576104 India; 2 Department of Emergency Medicine, Kasturba Medical College and Hospital, Manipal Academy of Higher Education, Manipal, Karnataka 576104 India; 3 Centre for Toxicovigilance and Drug Safety, Manipal College of Pharmaceutical Sciences, Manipal Academy of Higher Education, Manipal, Karnataka 576104 India

**Keywords:** Bacteria, Microbial susceptibility, Skin infection, Soft tissue infection, South Asia

## Abstract

**Background:**

Skin and soft tissue infections (SSTIs) have become a global public health threat especially in the Indian context. Secondary complications from untreated skin lesions make the patient life miserable and result in serious adverse effects. The main purpose of this scoping review is to identify the common bacterial pathogens causing SSTIs in South Asia.

**Methods:**

This scoping review adheres to the Joanna Briggs Institute (JBI) guidelines. The search strategy and screening follow the Peer Review of Electronic Search Strategies (PRESS) checklist, and the review will be reported according to Preferred Reporting Items for Systematic Reviews and Meta-Analyses Extension for Scoping Reviews (PRISMA-ScR) guidelines. The initial search set will follow the keyword such as “bacteria”, “microbial susceptibility”, and “South Asia”. Based on the predetermined inclusion and exclusion criteria, literature search will be carried out at the Cochrane Library, PubMed/MEDLINE, SCOPUS, Embase, CINAHL and Web of Science databases, relevant regional databases, and other grey literature will also be searched to cover maximum information. The data extraction and synthesis will be carried out by the researchers independently and the results will be summarised narratively.

**Discussion:**

The present scoping review seeks to address this gap by synthesizing evidence on the common bacterial pathogens causing SSTIs in children and adults across South Asia and their antibiotic susceptibility profiles. By contextualizing these findings, the review aims to inform region-specific empirical guidelines, ultimately enhancing clinical decision-making and improving patient outcomes in managing SSTIs in South Asia. In summary, the insights of this review are not only critical for improving clinical outcomes but also hold considerable public health significance by informing antimicrobial stewardship strategies and guiding policy efforts to address the growing challenge of antimicrobial resistance in the region.

**Scoping review Registration:**

This research has been registered in the Open Science Framework (OSF) (https://osf.io/xve49).

**Supplementary Information:**

The online version contains supplementary material available at 10.1186/s13643-025-02885-1.

## Background

Skin and soft tissue infections (SSTIs) are among the most common hospital-acquired infections (HAIs), following ventilator-associated pneumonia (VAP) and bloodstream infections (BSIs) [1]. The increasing rates of antibiotic resistance in South Asia have compounded the burden of SSTIs, highlighting the urgent need for evidence-based guidelines to support empirical antibiotic therapy [2]. Globally, the prevalence of SSTIs has ranged from 29 to 32% of all infections between 2018 and 2020 [3, 4]. The severity of these infections spans a spectrum—from minor superficial lesions that resolve with topical antibiotics to severe, life-threatening conditions necessitating surgical intervention and hospitalization [4]. Uncomplicated SSTIs, such as impetigo, carbuncles, furuncles, and superficial ulcers, can progress into more severe, complicated infections when subcutaneous or adjacent soft tissues become involved, as seen in cellulitis, necrotizing fasciitis, and complex ulcers. This progression is often exacerbated by comorbid conditions such as diabetes mellitus, ischemia, immunosuppression, or cell injury, which may also contribute to treatment failures [5–7]. Bacterial, fungal, and viral pathogens drive the pathogenesis of SSTIs, with *Staphylococcus aureus*, *Pseudomonas aeruginosa*, *Klebsiella pneumoniae*, *Escherichia coli*, and *Candida species* being the most frequently implicated [3, 7]. In resource-constrained clinical settings across South Asia, empirical antibiotic therapy is often initiated for uncomplicated infections while awaiting culture and sensitivity results [8]. However, the effectiveness of this approach is threatened by selection pressure and evolving bacterial resistance patterns, as evidenced by periodic changes in hospital antibiograms. This underscores the importance of antimicrobial stewardship programs, which aim to optimize antibiotic use, reduce treatment failures, and mitigate the impact of AMR [8–10].

Despite the significant burden of SSTIs and AMR in South Asia, there is a notable paucity of comprehensive reviews focusing specifically on the bacterial pathogens responsible for these infections and their antibiotic susceptibility profiles within this region. While global reviews highlight broader trends in antibiotic resistance, they often overlook the unique epidemiological patterns shaped by regional factors such as climate, hygiene practices, healthcare infrastructure, and antibiotic stewardship approaches [11, 12]. Existing global literature reviews, such as those by Napolitano et al. [5] and Dryden et al. [7], provide valuable insights into SSTIs but do not specifically address the South Asian context. Currently, there is no comprehensive scoping review that systematically examines the prevalence of common bacterial pathogens, their antibiotic susceptibility patterns, and regional resistance SSTIs—underscoring the need for such a review to inform evidence-based management in this region.

A systematic mapping of this evidence is essential to identify resistance trends, optimize treatment strategies, and inform antimicrobial stewardship programs tailored to the unique challenges of the region. By synthesizing the available data, this review will provide clinicians, researchers, and policymakers with a reliable reference to guide empirical antibiotic selection, strengthen AMR surveillance efforts, and develop targeted interventions to curb resistance. Thus, the present scoping review aims to synthesize evidence on the common bacterial pathogens causing SSTIs in children and adults across South Asia and their antibiotic susceptibility profiles. By contextualizing these findings, the review seeks to inform region-specific empirical guidelines, ultimately enhancing clinical decision-making and improving patient outcomes in managing SSTIs in South Asia.

## Objective

The objective of this scoping review is to identify and describe the common bacterial pathogens causing SSTIs in both children and adults across South Asia, along with their antimicrobial susceptibility profiles. The review will map existing evidence to inform surveillance efforts, guide empirical treatment strategies, and highlight regional resistance trends, with the aim of supporting antimicrobial stewardship and improving clinical outcomes.

## Methods

This scoping review will be conducted following the latest guidelines provided by the Joanna Briggs Institute (JBI) to comprehensively explore the literature on common bacterial pathogens causing SSTIs in children and adults across India and the broader South Asian context, with a specific focus on their antimicrobial susceptibility profiles [13]. Furthermore, this review will adhere to the Preferred Reporting Items for Systematic Reviews and Meta-Analyses Extension for Scoping Reviews (PRISMA-ScR) guidelines to ensure transparency, rigor, and comprehensive reporting throughout the review process [14]. Additionally, the study has been registered with the Open Science Framework (OSF) (https://osf.io/xve49).

The proposed review will be guided by the Arksey and O’Malley framework for systematic scoping reviews, which involves five key steps: (i) identifying the research question, (ii) conducting a relevant literature search, (iii) selecting studies, (iv) extracting data, and (v) summarizing and collating the results. Furthermore, a quality appraisal of the selected studies will be conducted, if necessary, to ensure the reliability and validity of the included studies [15].

### Step 1. Identifying the research question

The main research question is “What are the common bacterial organisms that cause skin lesions in children and adults in India and their antibiotic susceptibility profiles in India/South Asian countries?”.

The secondary research questions include the following:

i. What demographic factors (age, gender, etc.) are associated with different types of skin lesions in the studied populations?
ii. What are the most frequently prescribed empirical antibiotics for SSTIs in India, and how do these align with the susceptibility profiles of the identified bacterial organisms?
iii. What are the knowledge and practices of healthcare providers regarding the management of SSTIs and antibiotic prescribing?

The study will adopt a PO (Population, Outcome) format (Table 1) to align the research question with the study selection criteria.

**Table 1:** PO framework for the study selection and inclusion. Review Question: What are the common bacterial organisms that cause skin lesions in children and adults in India and their antibiotic susceptibility profiles in India/Indian subcontinent?

Framework	Description
Population	Patients with suspected infective skin lesion
Outcome	1. Prevalence of causative bacterial organism identified in pus culture/blood culture/others 2. Antimicrobial susceptibility

^*^Patients who have been administered antibiotics (oral or parenteral) for 24 h or more

### Step 2: Conducting a relevant literature search

In designing the search strategies for this scoping review, we balanced pragmatic considerations with scientific rigor. We selected databases such as PubMed, Scopus, Embase, Web of Science, CINAHL, and the Cochrane Library databases due to their relevance to biomedical literature, ensuring comprehensive coverage of infectious diseases, clinical microbiology, and antimicrobial resistance. These databases specialize in indexing studies on bacterial pathogens, antimicrobial susceptibility patterns, and treatment strategies, which are critical for this review. In addition, we will search in the available regional databases such as IndMed (India), MedIND (India), BanglaJOL (Bangladesh Journals Online), NepJOL (Nepal Journals Online), PakMediNet (Pakistan), and Sri Lanka Journals Online (SLJL) to ensure adequate representation of regional evidence and enhance comprehensive data retrieval, particularly for studies that may not be indexed in international databases. Additionally, we will consult official websites such as the International Clinical Trials Research Platform (ICTRP) and the Clinical Trials Registry-India (CTRI) to gather information on ongoing research.

Grey literature strategies, including searches of government reports and conference proceedings, to capture a complete picture of the available evidence, including studies not published in traditional journals. Institutional repositories, university theses, and digital repositories such as Shodhganga and policy briefs from major health agencies such as the World Health Organization Regional Office for South-East Asia (WHO-SEARO), Indian Council of Medical Research (ICMR), and the National Centre for Disease Control (NCDC) will also be searched to capture surveillance data on AMR trends, microbiology, and national health policies. To further enhance the comprehensiveness of our review and to enhance the inclusivity of our search strategy, we will employ a manual approach to capture studies with lower visibility including citation tracking (both forward and backward searches) and explore non-indexed sources such as Google Scholar and ResearchGate. Query strings will be tailored to each database using established frameworks like PICO to maximize relevant results while minimizing irrelevant ones such as “Skin Diseases,” “Skin Diseases, Infectious,” “Soft Tissue Infections,” “Drug Resistance, Microbial,” “Microbial Sensitivity Tests,” and “South Asia.” An example of the search strategy is given in the Supplementary file.

In addition to the primary search strategies for this scoping review, we will employ a multilingual search approach to enhance the comprehensiveness of our literature search. By exploring relevant literature published in regional languages, we aim to capture studies that may not be indexed in major databases but are essential for understanding the local context of skin and soft tissue infections in India and other South Asian countries. To ensure comprehensive coverage of relevant studies, this review will include literature published in languages other than English, particularly those in South Asian languages. Given the regional focus of this study, we will employ Microsoft Translator for translating non-English studies while maintaining contextual accuracy. The translation process will follow a structured approach, beginning with the initial screening of titles and abstracts using Microsoft Translator to determine relevance. If a study meets the inclusion criteria, full-text translation will be conducted using Microsoft Translator, ensuring consistency in language interpretation. To further improve accuracy, bilingual reviewers proficient in the respective languages will verify the translated content and cross-check for errors. This strategy will ensure our review is thorough and reflects the full spectrum of available evidence, ultimately enhancing the relevance and impact of our findings.

For this scoping review, we will also employ a search validation procedure to ensure the search strategy accurately retrieves relevant studies. We will first identify a test set of key articles known to be directly relevant to the research question, including landmark studies on SSTIs, bacterial organisms, and antibiotic susceptibility in South Asia. Using this set as a benchmardy selection and sc, we will conduct an initial trial search in each database and review whether the search captures all key articles. If any articles are missing, we will adjust search terms, Boolean operators, or field codes as needed—incorporating synonyms, alternative spellings, or modifying terms for broader or narrower scopes as required. After making modifications, we will re-run the adjusted search and confirm that it captures the entire test set. All adjustments and their rationales will be documented to ensure transparency and reproducibility of the search strategy. This iterative validation process will help maximize both the sensitivity and specificity of the search, ensuring comprehensive and relevant results.

Additionally, we will apply the Peer Review of Electronic Search Strategies (PRESS) checklist to systematically review and refine the search strategy [16]. The PRESS checklist will guide us in identifying potential issues and areas for improvement in the search terms, Boolean operators, and field selections. In addition to the checklist, we will consult a librarian with expertise in systematic reviews to refine the use of controlled vocabulary (e.g., MeSH terms, Emtree) and database-specific indexing strategies, ensuring a more precise and comprehensive search. This added layer of validation will further enhance the precision and reliability of the search.

### Step 3. Study selection and screening

The screening of titles and abstracts will be conducted according to the PO framework (Table 1). Additional selection criteria will guarantee that the content of the selected studies is following the research issue.

Before initiating the study selection process, all reviewers will undergo a structured training protocol to ensure consistency in applying the inclusion and exclusion criteria (Table 2). This training will include familiarization with the eligibility criteria and a pilot screening exercise on a subset of studies for blinded and independent selection.

**Table 2:** Study inclusion and exclusion criteria

Inclusion criteria	Exclusion criteria
Studies involving human populations with SSTIs in India or South Asia, across all age groups (children, adolescents, and adults)	Studies not conducted in India or South Asia or focusing on populations without SSTIs
Research assessing antibiotic use for SSTI treatment, including antimicrobial resistance patterns	Research unrelated to antibiotic treatment for SSTIs
Patients treated with antibiotics (oral or parenteral) for SSTIs for ≥ 24 h	Studies that do not report bacterial identification or antibiotic susceptibility
Studies identifying common bacterial organisms causing SSTIs and their antibiotic susceptibility (including partial antibiogram data)	Articles not fully accessible or available for review, unless efforts to retrieve them (e.g., author contact, institutional access) are unsuccessful. However, efforts will be made to access full texts through institutional access or contacting authors before excluding such articles
Observational studies (cross-sectional, cohort, case–control), surveillance studies, and clinical studies reporting microbiological or susceptibility data	
Studies published in English or regional languages (with translation and verification)	

The screening process for this scoping review will follow a two-stage approach to efficiently manage and evaluate the search results:

(i) Title and abstract screening: In the first stage, we will screen the titles and abstracts of the retrieved articles. Human reviewers will assess each entry for relevance to our review question based on our inclusion criteria. Articles that do not meet the criteria will be excluded at this stage.
(ii) Full text screening: In the second stage, we will perform a full-text review of the articles that passed the title and abstract screening. Human reviewers will comprehensively evaluate the content of each study to confirm its eligibility for inclusion in the review. Any articles that do not meet the established criteria will be excluded.

For this scoping review, additional search strategies will be employed to ensure comprehensive coverage of relevant studies. These include the ascendancy approach, where we will examine the reference lists of included studies to identify additional sources that may not have been captured by the primary search, thus uncovering foundational or related literature. Additionally, we will use the descendency approach through citation tracking tools like Google Scholar and CrossRef to find studies that cite our included sources, allowing us to capture more recent studies that reference key findings in the field.

Deduplication procedure: Throughout the screening process, we will implement a deduplication procedure to identify and remove any duplicate entries from the search results. This will be done using Microsoft Excel 2016 to streamline the process and maintain an accurate count of unique studies for screening.

In the screening process for the review, two independent screeners will conduct both the title and abstract screening, as well as the full-text screening, ensuring their assessments are made without prior discussion. During the title and abstract screening, both screeners will evaluate the studies independently, and the same procedure will apply during the full-text screening. To assess the reliability of their decisions, Cohen’s Kappa statistic will be used to measure the level of agreement between the screeners, with established Kappa value interpretations guiding further actions if poor agreement (Kappa < 0.40) is observed [17]. In such cases, the screeners will discuss any discrepancies and may include a third reviewer to reach a consensus.

### Step 4. Data extraction

The data from the primary research studies will be extracted using a validated data extraction form. Key elements for extraction, such as demographic details, outcome measures, and risk of bias indicators, will be carefully selected to address the research questions and support a comprehensive analysis. The extraction process will follow a multi-stage approach, starting with independent data extraction by two extractors, followed by a reconciliation phase to resolve any discrepancies. This two-step process enhances reliability by fostering discussion and consensus among extractors.

During the data extraction phase, two extractors will independently work on a predetermined set of studies to ensure unbiased extraction. Their outputs will be compared to identify discrepancies, which will then be resolved through discussion. In cases of significant differences, a senior researcher may be consulted to reach a consensus. The inter-rater reliability will be assessed using Cohen’s Kappa statistic, with a Kappa value above 0.70 considered acceptable, indicating strong agreement between extractors. This systematic method ensures the consistency and quality of the extracted data.

No masking will be applied during the data extraction process. Extractors will have full knowledge of the research questions, hypotheses, and the specific significance of the entities being extracted. This transparency allows them to understand the context and importance of the data, enhancing accuracy and relevance. Detailed protocols based on predefined inclusion and exclusion criteria will guide the extraction process, ensuring uniformity. These criteria will be communicated clearly to the extractors, and regular discussions will be held to address any questions or concerns. This open and systematic approach, supported by regular communication and adherence to detailed protocols, aims to ensure high-quality, contextually accurate data extraction that aligns with the review’s objectives.

### Step 5. Summarising and reporting the results

This scoping review aims to identify the common bacterial pathogens causing SSTIs and their antimicrobial susceptibility profile in South Asia. A narrative report will be produced to summarize the extracted data focusing primarily on the following outcomes: geographic region of study, age group (children or adults), prevalence of SSTIs, common bacterial pathogens identified, antibiotic susceptibility profiles, and factors associated with the occurrence and progression of SSTIs. The qualitative data will be analysed using thematic analysis to identify patterns and coherent themes aligned with the research questions. Findings will be contextualized within the broader objective of the review to inform region-specific empirical treatment strategies. Key themes will be inductively derived and coded manually. Where applicable, descriptive statistics (e.g., frequency counts, proportions) will be used to support the synthesis. The findings will be presented in text, tables, and figures to enhance clarity and accessibility. Additionally, the report will highlight gaps in the existing literature, such as regions within the South Asian countries with limited data on SSTIs or insufficient information on specific bacterial pathogens and their resistance patterns.

The methodological limitations of the included studies will be reported systematically to ensure transparency and critical appraisal of the findings. While formal critical appraisal is not mandatory for scoping reviews, we will conduct a descriptive assessment of study limitations, which will be presented in the Results and Discussion sections of the full review. To achieve this, we will extract and report key methodological concerns such as small sample sizes, study design biases, incomplete antibiogram reporting, lack of standard diagnostic criteria, and inconsistencies in antimicrobial susceptibility testing methods. These limitations will be summarized in a narrative synthesis, highlighting their potential impact on the interpretation of results. Additionally, we will categorize studies based on their design (e.g., cross-sectional, retrospective, prospective) and note methodological differences that may influence comparability.

This gap identification will provide insights into areas that require further research to support evidence-based management of SSTIs.

## Discussion

The proposed scoping review aims to identify and describe the common bacterial pathogens causing SSTIs in children and adults across India and the broader South Asian countries, along with their antimicrobial susceptibility profiles. It will also highlight gaps in the existing knowledge regarding the prevalence and resistance patterns of bacterial pathogens associated with SSTIs in this region.

This review represents the first step toward developing region-specific empirical guidelines for managing SSTIs. A comprehensive understanding of the bacterial pathogens and their susceptibility patterns will aid clinicians in selecting appropriate antibiotics, support antimicrobial stewardship programs, and inform public health strategies to combat AMR. These findings may also contribute to updating existing national and regional treatment guidelines for SSTIs, ensuring that empirical therapy aligns with current resistance trends and is adapted to the local epidemiological context. Furthermore, the findings may create awareness about the burden of SSTIs and their associated challenges, guiding healthcare policymakers and stakeholders to address these issues effectively.

A limitation of this review is that since studies will not undergo a formal quality appraisal, the reliability of data extracted from the selected studies cannot be fully assessed. These limitations could affect the comprehensiveness and generalizability of the findings.

## Supplementary Information

Additional file 1: Supplementary file.

## Data Availability

All data generated or analysed during this study will be included in the published scoping review article.

## Abbreviations

SSTI: Skin and soft tissue infection
HAI: Hospital-acquired infections
CINAHL: Cumulative Index to Nursing and Allied Health Literature
VAP: Ventilator-associated pneumonia
BSI: Bloodstream infection
AMR: Antimicrobial resistance
MDR: Multidrug resistant
JBI: Joanna Briggs Institute
PRISMA-ScR: Preferred Reporting Items for Systematic Reviews and Meta-Analyses Extension for Scoping Reviews
OSF: Open Science Framework
ICTRP: International Clinical Trials Research Platform
CTRI: Clinical Trials Registry-India
PRESS: Peer Review of Electronic Search Strategies
WHO-SEARO: World Health Organization Regional Office for South-East Asia
ICMR: Indian Council of Medical Research
NCDC: National Centre for Disease Control
BanglaJOL: Bangladesh Journals Online
NepJOL: Nepal Journals Online
SLJL: Sri Lanka Journals Online
