# Supplementary material for: Bacterial pathogens causing skin and soft tissue infections and antibiotic susceptibility in South Asia: a scoping review protocol
Source: Syst Rev. 2025 Jul 4;14:136. doi: 10.1186/s13643-025-02885-1 (PMC12231741; doi:10.1186/s13643-025-02885-1)
Supplement: Supplementary file 1 — Additional file 1: Supplementary file. [file 13643_2025_2885_MOESM1_ESM.docx]

| **Search** | **Query** |
| --- | --- |
| #1 | (((Skin Disease) OR (Dermatosis)) OR (Dermatoses)) OR (Skin and Subcutaneous Tissue Disorders) |
| #2 | (((Bacterial Skin Disease) OR (Disease, Bacterial Skin)) OR (Skin Disease, Bacterial)) OR (Bacterial Skin Diseases) |
| #3 | ((((Infectious Skin Diseases) OR (Disease, Infectious Skin)) OR (Diseases, Infectious Skin)) OR (Infectious Skin Disease)) OR (Skin Disease, Infectious) |
| #4 | Phlegmon |
| #5 | Abscesses |
| #6 | ((Folliculitides) OR (Sycosis)) OR (Sycoses) |
| #7 | ((((Impetigos) OR (Impetigo Contagiosa)) OR (Contagiosa, Impetigo)) OR (Contagiosas, Impetigo)) OR (Impetigo Contagiosas) |
| #8 | ((((((Fasciitides, Necrotizing) OR (Necrotizing Fasciitides)) OR (Necrotizing Fasciitis)) OR (Fascitis, Necrotizing)) OR (Fascitides, Necrotizing)) OR (Necrotizing Fascitides)) OR (Necrotizing Fascitis) |
| #9 | ((Infections, Soft Tissue) OR (Infection, Soft Tissue)) OR (Soft Tissue Infection) |
| #10 | ((((((((#1) OR (#2)) OR (#3)) OR (#4)) OR (#5)) OR (#6)) OR (#7)) OR (#8)) OR (#9) |
| #11 | Eubacteria |
| #12 | (((((((((((Drug Resistances, Microbial) OR (Antibiotic Resistance, Microbial)) OR (Antibiotic Resistance)) OR (Resistance, Antibiotic)) OR (Antimicrobial Drug Resistance)) OR (Antimicrobial Drug Resistances)) OR (Antimicrobial Resistance, Drug)) OR (Antimicrobial Resistances, Drug)) OR (Drug Antimicrobial Resistance)) OR (Drug Antimicrobial Resistances)) OR (Resistance, Drug Antimicrobial)) OR (Resistances, Drug Antimicrobial) |
| #13 | (((((((((((((((((((((((((((Microbial Sensitivity Test) OR (Sensitivity Test, Microbial)) OR (Sensitivity Tests, Microbial)) OR (Test, Microbial Sensitivity)) OR (Tests, Microbial Sensitivity)) OR (Drug Sensitivity Assay, Microbial)) OR (Minimum Inhibitory Concentration)) OR (Concentration, Minimum Inhibitory)) OR (Concentrations, Minimum Inhibitory)) OR (Inhibitory Concentration, Minimum)) OR (Inhibitory Concentrations, Minimum)) OR (Minimum Inhibitory Concentrations)) OR (Virus Drug Sensitivity Tests)) OR (Viral Drug Sensitivity Tests)) OR (Fungus Drug Sensitivity Tests)) OR (Fungal Drug Sensitivity Tests)) OR (Antibiogram)) OR (Antibiograms)) OR (Antimicrobial Susceptibility Breakpoint Determination)) OR (Breakpoint Determination, Antimicrobial Susceptibility)) OR (Bacterial Sensitivity Tests)) OR (Bacterial Sensitivity Test)) OR (Sensitivity Test, Bacterial)) OR (Sensitivity Tests, Bacterial)) OR (Test, Bacterial Sensitivity)) OR (Tests, Bacterial Sensitivity)) OR (Breakpoint Determination, Antibacterial Susceptibility)) OR (Antibacterial Susceptibility Breakpoint Determination) |
| #14 | ((((((((((((((((((((((((((((((((Agents, Anti-Bacterial) OR (Anti Bacterial Agents)) OR (Anti-Bacterial Compounds)) OR (Anti Bacterial Compounds)) OR (Compounds, Anti-Bacterial)) OR (Antibacterial Agents)) OR (Agents, Antibacterial)) OR (Anti-Bacterial Agent)) OR (Agent, Anti-Bacterial)) OR (Anti Bacterial Agent)) OR (Anti-Bacterial Compound)) OR (Anti Bacterial Compound)) OR (Compound, Anti-Bacterial)) OR (Antibacterial Agent)) OR (Agent, Antibacterial)) OR (Antibiotics)) OR (Antibiotic)) OR (Anti-Mycobacterial Agents)) OR (Agents, Anti-Mycobacterial)) OR (Anti Mycobacterial Agents)) OR (Antimycobacterial Agents)) OR (Agents, Antimycobacterial)) OR (Anti-Mycobacterial Agent)) OR (Agent, Anti-Mycobacterial)) OR (Anti Mycobacterial Agent)) OR (Antimycobacterial Agent)) OR (Agent, Antimycobacterial)) OR (Bacteriocidal Agents)) OR (Agents, Bacteriocidal)) OR (Bacteriocides)) OR (Bacteriocidal Agent)) OR (Agent, Bacteriocidal)) OR (Bacteriocide) |
| #15 | ((#11) OR (#12)) OR (#13)) OR (#14) |
| #16 | (((Asia, South) OR (South Asia)) OR (Southern Asia)) OR (British Indian Ocean Territory) |
| #17 | ((#10) AND (#15)) AND (#16) |
